# Supplementary material for: Lung cancer-enriched p53 mutants occupy canonical p53 target genes without activating transcription, revealing a distinct loss-of-function behavior
Source: bioRxiv. 2026 Feb 17:2026.02.14.705936. Preprint. [Version 1] doi: 10.64898/2026.02.14.705936 (PMC12934569; doi:10.64898/2026.02.14.705936)
Supplement: Supplement 1 [file media-1.pdf]

Name	Sequence	Application
GAPDH FWD	TGGGCTACACTGAGCACCAG	RT-qPCR
GAPDH REV	GGGTGTCGCTGTTGAAGTCA	RT-qPCR
p21 FWD	TGTCCGTCAGAACCCATGC	RT-qPCR
p21 REV	AAAGTCGAAGTTCCATCGCTC	RT-qPCR
RRM2B FWD	ATTGGGCCTTGCGATGGATAG	RT-qPCR
RRM2B REV	GAGTCCTGGCATAAGACCTCT	RT-qPCR
PLK3 FWD	TTTTCGCACCACTTTGAGGAC	RT-qPCR
PLK3 REV	GAGGCCAGAAAGGATCTGCC	RT-qPCR
BAX FWD	CCCGAGAGGTCTTTTTCCGAG	RT-qPCR
BAX REV	CCAGCCCATGATGGTTCTGAT	RT-qPCR
PUMA FWD	GACCTCAACGCACAGTACGAG	RT-qPCR
PUMA REV	AGGAGTCCCATGATGAGATTGT	RT-qPCR

Supplemental Table 1: List of primers used in RT-qPCR analyses.

Name	Sequence	Application
p21 RE FWD	CTCCATCCCTATGCTGCCTG	ChIP
p21 RE REV	AGGCAGCCCAAGGACAAAAT	ChIP
PLK3 RE FWD	ACCCGAAATTTGCCCCTCAA	ChIP
PLK3 RE REV	CTGCCTTGCCAGGTTTAGGA	ChIP
RRM2B RE FWD	TGGGCATGTCAGGACAACAA	ChIP
RRM2B RE REV	CCAGCCTAGATTGGGGCATC	ChIP
BAX RE FWD	TGCGATCTCCAAGCACTGAG	ChIP
BAX RE REV	CTTCCGAGATCCCCTGGTTC	ChIP
PUMA RE FWD	GAACCCCACACAAACAGGC	ChIP
PUMA RE REV	GTGGTGGTGTCGATCCAGAG	ChIP

Supplemental Table 2: List of primer sequences used in ChIP-qPCR analyses.

Name	Sequence	Application
p21 FWD	5BiosG/ATGATGATGAACATGTCCCAACATGTTGATATGATG	SPR
p21 REV	CATCATATCAACATGTTGGGACATGTTCATCATCAT	SPR
BAX FWD	5BiosG/ATGATGATGGGCAGGCCCGGGCTTGTCGATATGATG	SPR
BAX REV	CATCATATCGACAAGCCCGGGCCTGCCCATCATCAT	SPR
Scrambled FWD	5BiosG/ATGATGATATGAGCACTGTCGTCATAACATATGATG	SPR
Scrambled REV	CATCATATGTTATGACGACAGTGCTCATATCATCAT	SPR
AEN FWD	5BiosG/ATGATGATGGGCTTGCCCGGGCATGTGGATATGATG	SPR
AEN REV	CATCATATCCACATGCCCGGGCAAGCCCATCATCAT	SPR
PLK3 FWD	5BiosG/ATGATGATTAAACATGCCCGGGCAAAAGCATATGATG	SPR
PLK3 REV	CATCATATGCTTTTGCCCGGGCATGTTTAATCATCAT	SPR
PUMA FWD	5BiosG/ATGATGATGGACAAGTCAGGACTTGCAGATATGATG	SPR
PUMA REV	CATCATATCTGCAAGTCCTGACTTGTCCATCATCAT	SPR

Supplemental Table 3: List of DNA sequences used in SPR analyses.

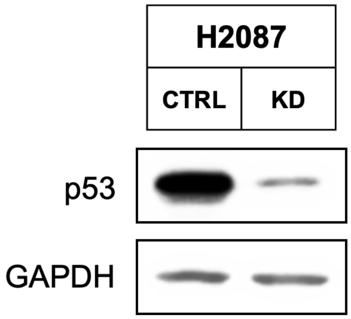

Supplemental Figure 1: p53 was successfully depleted in the KD condition.
Immunoblot of GADPH and p53 proteins using whole cell lysates collected from H2087 cells and H2087 p53 KD cells used for the control condition in Figure 1A.

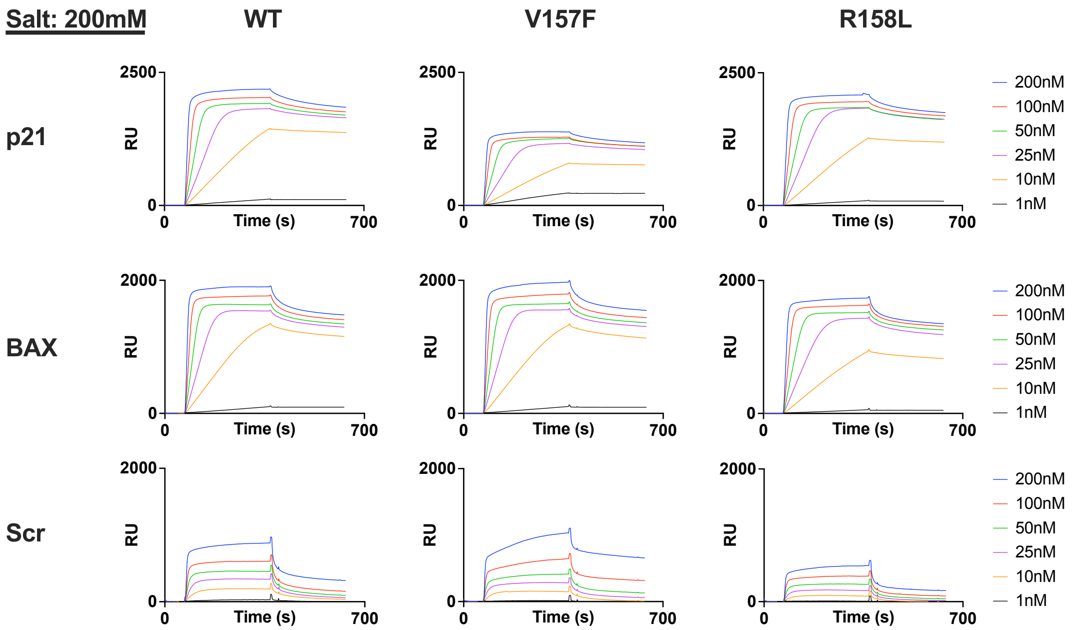

Supplemental Figure 2: V157F and R158L p53 mutants bind to p53 target genes p21, BAX, and Scramble similar to WT p53 at 200mM salt by SPR.

SPR binding data in RU for WT, V157F, and R158L p53 at protein concentrations of 1nM-200nM and salt concentration of 200mM. Binding was checked using p21 and BAX response elements and a scrambled control DNA sequence (Scr).

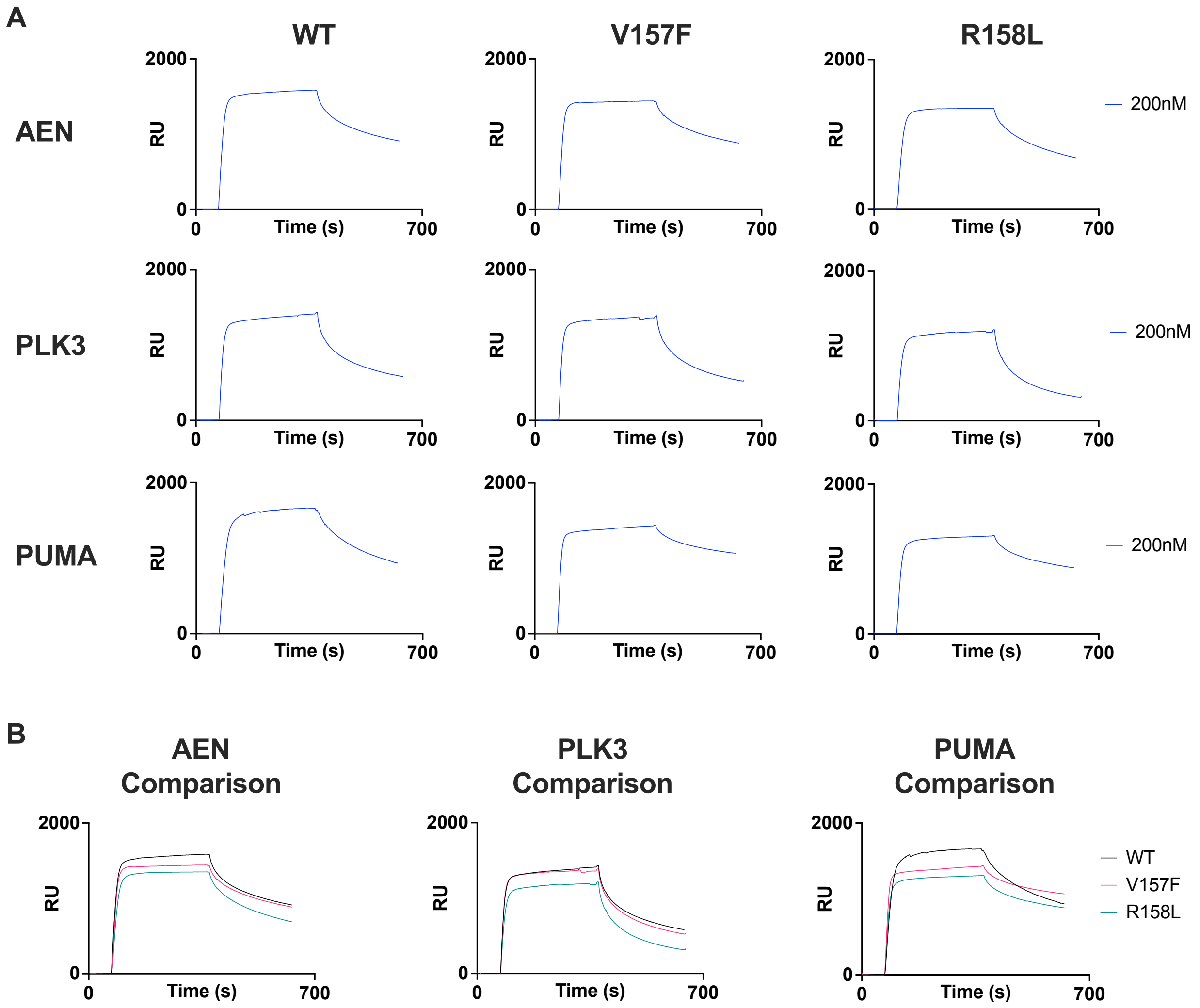

Supplemental Figure 3: V157F and R158L p53 mutants bind to p53 target genes AEN, PLK3, and PUMA similar to WT p53 at 275mM salt by SPR.

A, SPR binding data in RU for WT, V157F, and R158L p53 at 200nM protein concentration and a salt concentration of 275mM. Binding was checked using AEN, PLK3, and PUMA response elements. **B,** Comparisons between WT and mutant p53 displaying the data from (H) for AEN, PLK3, and PUMA response elements.

	WT p53	V157F p53	R158L p53
Salt Concentration:	275mM	275mM	275mM
	KD (nM)	KD (nM)	KD (nM)
AEN	7.14	5.83	11.50
PLK3	10.10	10.50	21.90
PUMA	2.83	3.15	5.33

Supplemental Table 4: KD values derived from SPR data for WT p53 and mutant p53 binding interactions with DNA sequences for AEN, PLK3, and PUMA at 275mM salt concentration.

	WT p53					R158L p53					V157F p53			
**Glutaraldehyde %**	0	0.0<br>01	0.0<br>05	0.1		0	0.0<br>01	0.0<br>05	0.1		0	0.0<br>01	0.0<br>05	0.1

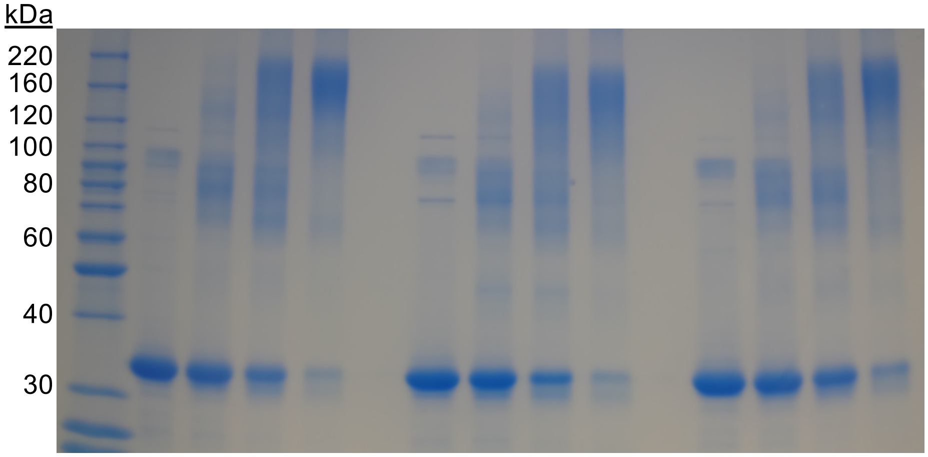

Supplemental Figure 4: V157F and R158L p53 mutants are able to form tetramers.
Purified p53 proteins (WT p53, R158L, or V157F p53) were cross-linked with differing concentrations of glutaraldehyde and run on a Coomassie gel.

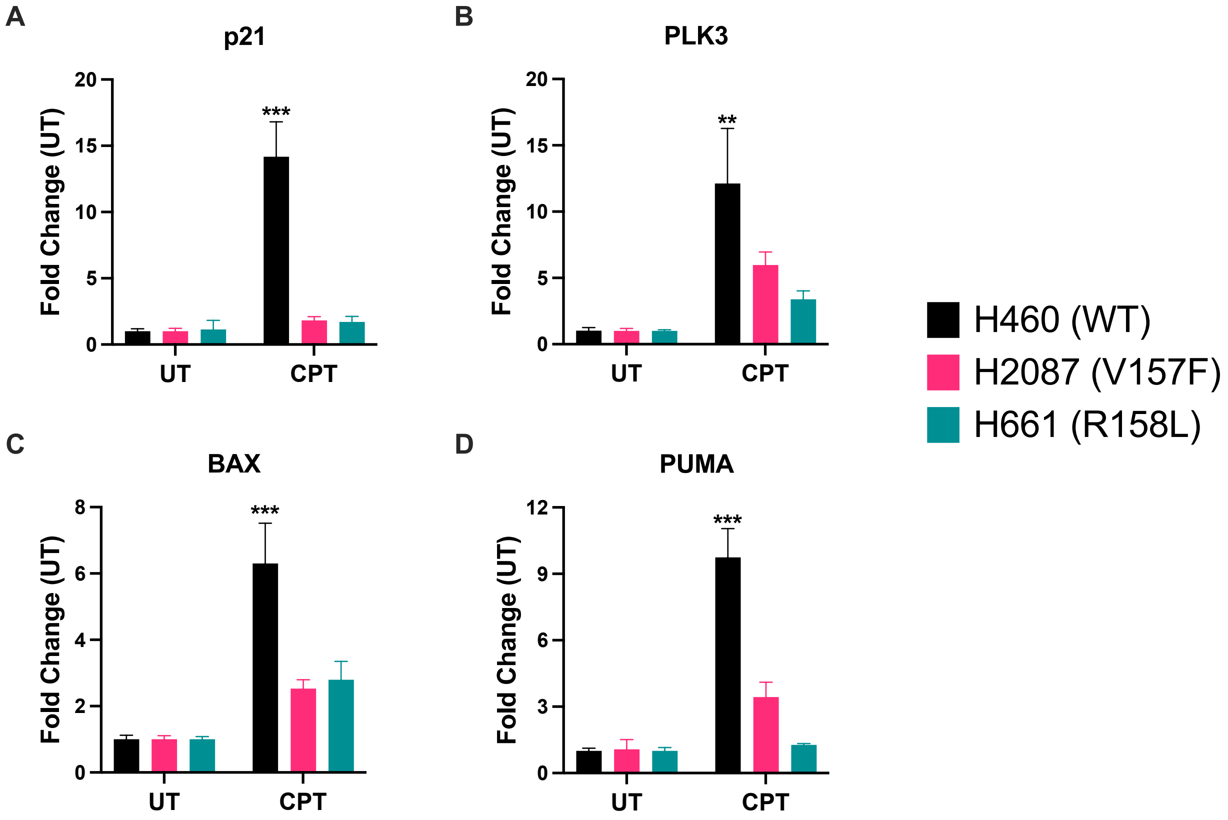

Supplemental Figure 5: Endogenously expressing V157F (H2087) and R158L (H661) p53 mutant cells do not induce p53 target gene expression to the same degree as WT p53 (H460) cells in response to CPT treatment.

A-D, H460 (WT p53), H2087 (V157F p53), and H661 (R158L p53) cells were treated with CPT (2μM) for 16 hours or left untreated. RNA was collected from the cells, and cDNA was created. RT-qPCR was performed on the samples utilizing primers for p21, PLK3, BAX, and PUMA. Significance is shown for H460 (WT) CPT-treated compared to H2087 (V157F) CPT and H661 (R158L) CPT, Two-way ANOVA, *** $p < 0.001$, ** $p < 0.01$.

	H1299 (WT p53)			H1299 (V157F p53)			H1299 (R158L p53)		
**Cell Cycle**	**TET**	**24hr TET/CPT**	**48hr TET/CPT**	**TET**	**24hr TET/CPT**	**48hr TET/CPT**	**TET**	**24hr TET/CPT**	**48hr TET/CPT**
Sub G1 (%)	1.6	7.8	29.7	1.5	0.7	7.8	1.2	1.6	14.5
G1 (%)	67.9	47.9	37.5	62.7	37.0	38.5	61.4	27.4	14.5
S (%)	17.4	21.3	13.3	18.5	42.3	17.4	18.5	51.5	11.9
G2/M (%)	12.3	20.6	18.9	15.7	17.4	34.7	17.7	17.5	53.7

Supplemental Table 5: Percentage of cells in each stage of the cycle in Figure 6A.

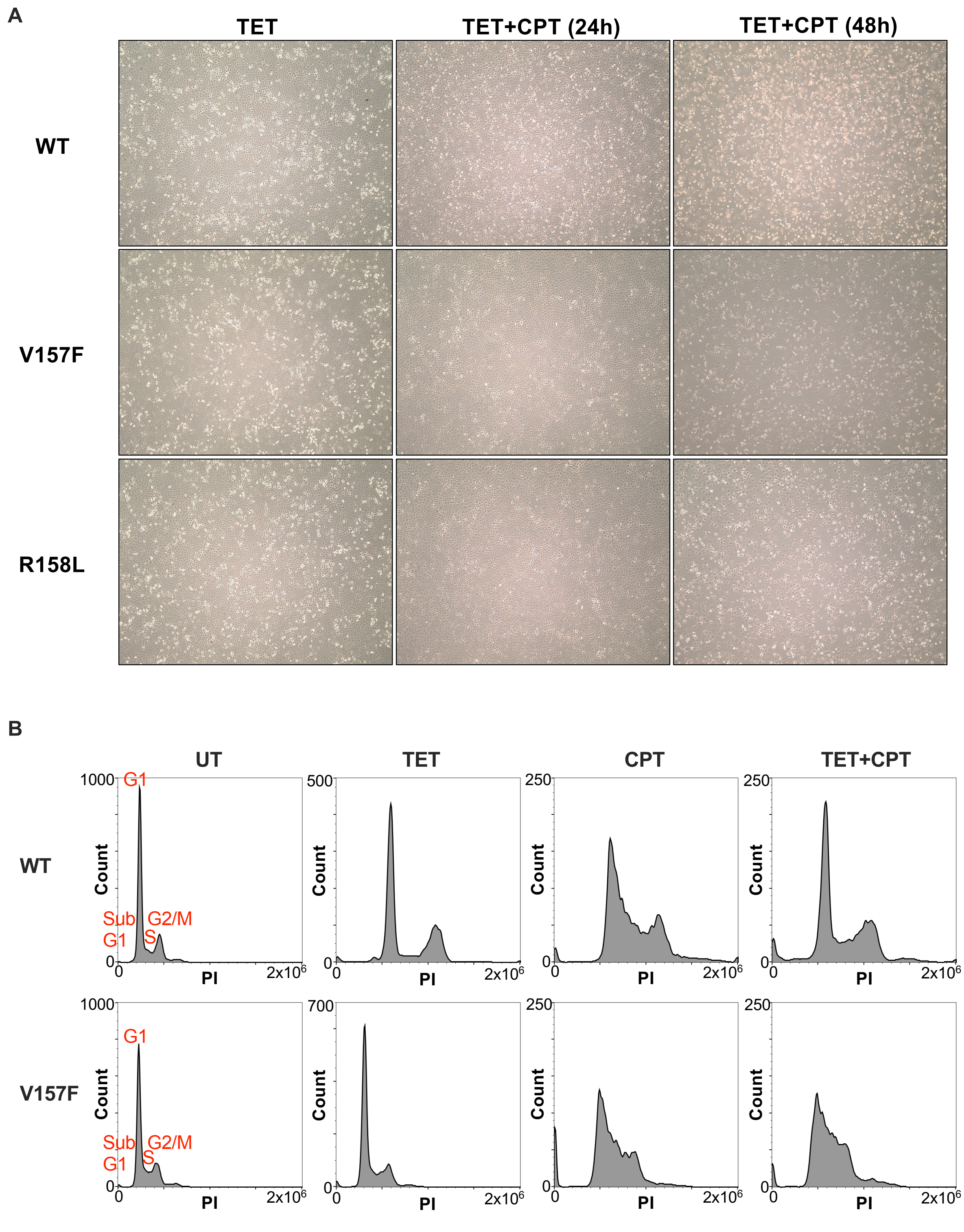

Supplemental Figure 6: V157F and R158L p53 mutants have a dominant-negative phenotype.

A, Representative brightfield images of H1299 cells used in Figure 6A. Scale bar = 350μm, 4X magnification. **B,** H1299 cells with a TET-inducible plasmid for WT or V157F p53 were treated for 24 hours with TET (1μM) alone, CPT (2μM) alone, TET(1μM) in combination with CPT (2μM), or left untreated. Cells were collected, and cell cycle analysis by flow cytometry was performed. Data analysis and histogram creation were done in FlowJo (10.10.0). Two-way ANOVA, $p < 0.05$.
